# Case Report: A case report and literature review of shwachman-diamond syndrome concurrent with klinefelter syndrome

**DOI:** 10.3389/fped.2025.1671169

**Published:** 2025-11-26

**Authors:** Chenyang Chang, Hao Chen, Hao Zhang, Jingshan Chen, Qinxin Wan, Huifang Zhu, Kaiyuan Luo, Xingyu Rao

**Affiliations:** 1 Pediatric Internal Medicine, Children’s Medical Center, First Affiliated Hospital of Gannan Medical University, Ganzhou, Jiangxi, China; 2 The First Clinical Medical College of Gannan Medical University, Ganzhou, Jiangxi, China; 3 Pediatric Intensive Care Unit, Fuqing City Hospital of Fujian, Fuzhou, Fujian, China; 4 Neonatal/Pediatric Intensive Care Unit, Children’s Medical Center, First Affiliated Hospital of Gannan Medical University, Ganzhou, Jiangxi, China; 5 Institute of Children’s Medical, First Affiliated Hospital of Gannan Medical University, Ganzhou, Jiangxi, China; 6 Ganzhou Key Laboratory of Immunotherapeutic Drugs Developing for Childhood Leukemia, Ganzhou, Jiangxi, China; 7 Basic Medical College of Gannan Medical University, Ganzhou, Jiangxi, China

**Keywords:** shwachman-Diamond syndrome, *SBDS* gene, Klinefelter syndrome, case report, mosaic pattern

## Abstract

Shwachman-Diamond syndrome (SDS) is a rare genetic disorder characterized by pancreatic insufficiency, metaphyseal chondrodysplasia, and bone marrow failure. These clinical features collectively contribute to the multisystemic nature of SDS, affecting multiple organ systems. In contrast, Klinefelter syndrome is defined by the presence of an additional X chromosome. Its clinical presentation primarily includes an abnormal testicular microenvironment, impaired spermatogenesis, decreased testosterone levels, and elevated gonadotropin levels. We identified a pediatric patient presenting with SDS concomitantly diagnosed with Klinefelter syndrome, characterized by a splice-site in the Shwachman-Bodian-Diamond Syndrome (*SBDS*) gene and a mosaic karyotype of 47,XXY/46,XY(Klinefelter syndrome). A 6-month-old infant was admitted to the hospital with elevated liver enzymes and neutropenia persisting for more than two weeks. Additional investigations revealed granulocytopenia, increased liver enzyme levels, and reduced fecal elastase, raising strong suspicion of SDS. Whole exome sequencing (WES) was conducted on the proband and both parents, revealing a homozygous variant in the *SBDS* gene (c.258+2T>C) located on chromosome 7 in the proband. Concurrently, the karyotype analysis demonstrated a mosaic pattern consistent with 47,XXY/46,XY(Klinefelter syndrome). The objective of this study is to improve the understanding of SDS and Klinefelter syndrome through a detailed analysis of their clinical manifestations and genetic profiles. This work aims to establish a solid molecular basis for etiological diagnosis, genetic counseling, and prenatal diagnosis of these syndromes.

## Introduction

Shwachman-Diamond syndrome (SDS) is a rare hereditary disorder characterized by defective ribosome biosynthesis (1). With an estimated incidence of approximately 1 in 76,000 live births, it demonstrates a male-to-female ratio of 1.7:1 (2, 3). The condition is multisystemic, presenting with a spectrum of clinical manifestations, primarily including pancreatic exocrine insufficiency, skeletal abnormalities, and bone marrow dysfunction (4). Patients with SDS exhibit an elevated risk of developing myelodysplastic syndrome (MDS) and acute myeloid leukemia (AML) (5). Genetic analyses have identified pathogenic variants in the Shwachman-Bodian-Diamond Syndrome (*SBDS*) gene in approximately 90% of diagnosed cases (6). The *SBDS* variants c.183_184TA>CT and c.258+2T>C are the most prevalent variants associated with SDS (7). However, homozygous variants of c.183_184TA>CT have not yet been reported, attributed to their almost complete lethality; in contrast, homozygous variants of c.258+2T>C have been documented (4, 8, 9). Nonetheless, the compound heterozygous variants involving both c.183_184TA>CT and c.258+2T>C is more commonly observed (10). Additionally, genes such as *EFL1, DNAJC21, and SRP54* have been implicated in the pathogenesis and progression of SDS (11–13). Klinefelter syndrome is the most prevalent chromosomal disorder in males, characterized by microorchidism, gynecomastia, hypogonadism, and elevated gonadotropin levels (14). This condition results from an additional X chromosome, leading to a range of physiological and hormonal abnormalities that significantly impact male reproductive health. The most prevalent karyotype associated with this disease is 47, XXY (Klinefelter syndrome), which accounts for approximately 80%–90% of cases; additionally, other chromosomal configurations include mosaic types such as 47,XXY/46,XY (Klinefelter syndrome), and rarer karyotypes, including 48,XXXY, 48,XXYY, and 47,iXq,Y, collectively representing the remaining 10%–20% (15). A Danish study reported an incidence rate of 150 per 100,000 individuals, corresponding to approximately 1 in 667 men (16). However, there is currently a paucity of comparable epidemiological data concerning the prevalence of this disease in our country.

We present a rare case of SDS resulting from a homozygous in the *SBDS* gene, co-occurring with mosaic 47,XXY/46,XY Klinefelter syndrome. Through a comprehensive analysis of the patient's clinical and genetic characteristics, along with a review of pertinent literature, this study seeks to improve clinical recognition of this complex disorder and minimize the potential for diagnostic oversight or error.

## Case description

This study received approval from the Ethics Committee of the First Affiliated Hospital of Gannan Medical University (LLSC-2025 No. 037), and informed consent was obtained from the child's guardian.

The patient was a 6-month-old male infant admitted to the hospital for evaluation of “abnormal liver enzyme levels and neutropenia persisting for more than two weeks.” Approximately two weeks prior to admission, the infant developed a febrile episode following vaccination. Subsequent laboratory investigations revealed elevated aminotransferase levels and neutropenia. He was initially admitted to a local hospital, where he received hepatoprotective therapy with compound glycyrrhizin and glutathione, along with antiviral treatment using acyclovir. Although the aminotransferase levels showed some reduction compared to the initial values, they failed to normalize. After discharge, the patient was maintained on oral compound glycyrrhizin. Upon re-evaluation, persistent elevation in transaminase levels was noted, leading to his referral to our institution. A detailed review of the medical history indicated that both elevated aminotransferases and neutropenia were present concurrently at initial presentation; however, the granulocytopenia had been overlooked and remained unaddressed during the prior management. The patient had developed an erythematous rash on both cheeks shortly after birth and had a known history of constipation. There were no significant abnormalities in the past medical history, personal history, or family history. His parents were non-consanguineous, and his two older sisters exhibited normal phenotypes. On physical examination, vital signs were stable. The infant's weight was 8.3 kg and length was 67 cm. He was alert and in a stable mental condition, with a flat anterior fontanelle. Skin turgor was good, and scattered erythematous macules were observed over both cheeks. Cardiopulmonary and abdominal examinations were unremarkable. Limb muscle strength and tone were normal, physiological reflexes were present, and no pathological reflexes were elicited. External genitalia appeared normal.

Upon admission, routine blood tests and liver function evaluations were conducted repeatedly, with the results summarized in Table 1. Additional auxiliary examinations were presented in Table 2.

**Table 1:** Results of complete blood count and liver function tests upon hospital admission.

Parameter	Result (d1)	Result (d4)	Result (d12)	Result (d15)	Reference range	Unit
Complete blood count test						
WBC	4.57	5.25	6.33	6.5	4.8–14.6	10^9^ /L
HGB	109	99	120	115	97–141	g/L
PLT	320	297	505	539	190–579	10^9^ /L
NEUT	0.33	0.82	0.81	0.4	0.8–6.4	10^9^ /L
Liver function assessment						
ALT	–	163	201	137	8–71	U/L
AST	–	133	153	125	21–80	U/L
TBA	–	19.1	31.7	16.1	<9.7	Umol/L

**Table 2:** The results of the additional auxiliary examinations.

Category	Examination items	Result	Reference range
Anomalous Results	Immunoglobulin E	237 IU/ml	≤15 IU/ml
	Cytomegalovirus IgG	26.126 AU/ml	<10 AU/ml
	Alpha-fetoprotein	208 ng/ml	0–7 ng/ml
	Anti-cytoplasmic antibodies	positive	–
	Amylase	19 U/L	35–135 U/L
	vitamin A	182.44 ng/mL	200–570 ng/mL
	Pancreatic elastase 1	70 ug/g	>200 ug/g
	Urine metabolic profiling	Adipic acid-2, octamethylene acid-2, and 3-hydroxymaleic acid-3.	–
	Bone marrow cytology examination	4% prolymphocytes	–
	Bilateral hip joint anteroposterior and lateral views	He left femoral head epiphysis was smaller in size compared to the right	–
Normal result	Electrolytes, renal function, myocardial enzymes	No significant abnormalities detected	–
	Immunoglobulins (IgG, IgM, IgA)	No significant abnormalities detected	–
	Complement components (C3, C4)	No significant abnormalities detected	–
	Ceruloplasmin	No significant abnormalities detected	–
	Rheumatoid factor	No significant abnormalities detected	–
	Cytomegalovirus IgM antibody	No significant abnormalities detected	–
	Toxoplasma gondii antibody, rubella virus antibody, herpes simplex virus antibody	No significant abnormalities detected	–
	Ferritin	No significant abnormalities detected	–
	Stool examination	No significant abnormalities detected	–
	Cytomegalovirus DNA (blood and urine)	No significant abnormalities detected	–
	Epstein-barr virus DNA	No significant abnormalities detected	–
	Autoimmune liver disease antibodies	No significant abnormalities detected	–
	Blood ammonia	No significant abnormalities detected	–
	Lactic acid	No significant abnormalities detected	–
	Lipid profile	No significant abnormalities detected	–
	Anti-ENA antibodies	No significant abnormalities detected	–
	Coagulation analysis	No significant abnormalities detected	–
	Lipase	No significant abnormalities detected	–
	Vitamin D, vitamin E, vitamin K	No significant abnormalities detected	–
	Blood metabolic screening	No significant abnormalities detected	–
	Doppler ultrasound of the digestive system, echocardiography, bilateral hip joint,	No significant abnormalities detected	–
	Unenhanced upper abdominal MRI	No significant abnormalities detected	–
	The five panels of hepatitis B, hepatitis classification, and thyroid function tests	No significant abnormalities detected	–

SDS was suspected based on the clinical manifestations, laboratory findings, and imaging results. To further investigate this hypothesis, Whole exome sequencing (WES) was performed using peripheral blood samples from both parents and the child. Genetic testing identified a homozygous variant (c.258+2T>C) in the *SBDS* gene located on chromosome 7. The results of Sanger sequencing indicated that the c.258+2T>C variant was inherited from the child's father and mother, respectively, consistent with an autosomal recessive inheritance pattern. In accordance with the guidelines established by the American College of Medical Genetics and Genomics (ACMG), the variant c.258+2T>C is classified as pathogenic. The genomic copy number variation sequencing (CNV-seq) based on next-generation sequencing (NGS) technology indicates the presence of 47,XXY/46,XY (Klinefelter syndrome) variants. It reveals a mosaic duplication of the X chromosome with an approximate mosaicism ratio of 53%, while the Y chromosome exhibits a copy number of 1. However, due to family-related circumstances pertaining to the affected child, chromosomal karyotype analysis was not performed. Currently, the child is undergoing treatment with compound glycyrrhizin for liver protection, granulocyte colony-stimulating factor (G-CSF) to increase granulocyte counts, and pancreatin powder.

## Discussion

SDS is a rare autosomal recessive genetic disorder characterized by biallelic variants in the *SBDS* gene in the majority of affected individuals (7, 17). The gene, situated on the long arm of chromosome 7 and comprising 5 exons spanning 7.9 kilobases, encodes a predicted protein consisting of 250 amino acids (7). As a cofactor of GTPase *EFL1, SBDS* plays an essential role in facilitating the release of eIF6 from the 60S ribosomal subunit and promoting the assembly of functional 80S ribosomes (18). It has been demonstrated that the pivotal function of *SBDS* is to tightly couple the activation of GTP hydrolysis with the release of eIF6 from the ribosome (19). Additionally, it has been confirmed that the absence of *SBDS* results in impaired ribosomal subunit joining, causing the process to stall at the pre-60S subunit stage (19). In patients with SDS, defective ribosome maturation leads to impaired protein synthesis, diminished hematopoietic stem cell function, elevated TP53 activity, and the activation of cellular checkpoint mechanisms (20). The *SBDS* variants c.183_184TA>CT and c.258+2T>C are the most prevalent variants associated with SDS (7, 10, 18). Both of these variants arise from gene conversion events involving the adjacent pseudogene SBDS protein (SBDSP), leading to a significant reduction or complete loss of functional SBDSP expression (7).

**Figure 1:**
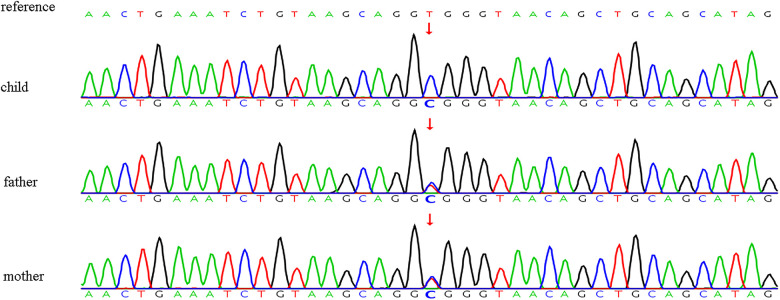
Schematic representation of the validation results obtained through sanger sequencing of *SBDS* variants in the child and his parents. SBDS, Shwachman-Bodian-Diamond Syndrome.

Genetic analysis in the present study identified a homozygous variant in the SBDS gene (c.258+2T>C) in the affected child. This variant is a canonical splice-site variant that disrupts the 5′ splice site of intron 2, leading to aberrant splicing and consequent alteration of protein function (7). Sanger sequencing confirmed that the c.258+2T>C variant was inherited from both parents, with each parent being a heterozygous carrier of the variant (Figure 1). In accordance with the ACMG guidelines, this variant had been classified as a pathogenic variant (PVS1 + PM3_VeryStrong). This variation, located within the splicing region, led to alterations in the function of the encoded protein, classifying it as a highly pathogenic variant (PVS1). In recessive genetic disorders, pathogenic or likely pathogenic variants were identified in the trans configuration relative to the reference allele (PM3_VeryStrong). The variant was absent in the Shenzhou population database but was reported at low allele frequencies in the 1000 Genomes Project (0.00159744), the Exome Aggregation Consortium (0.00394583), and the Genome Aggregation Database (0.00541586), consistent with its pathogenic status and carrier frequency in the general population.

The clinical manifestations of SDS are well-documented and highly heterogeneous, involving multiple organ systems such as the skeletal, cardiovascular, cutaneous, hepatic, immune, hematologic, and central nervous systems (1). Neutropenia, defined as an absolute neutrophil count below 1.5*10^9^ /L, is the most common hematological abnormality in SDS. A North American study reported that 81% of SDS patients presented with neutropenia (8), which was also one of the initial clinical features observed in the child described in this study. Additionally, patients may develop anemia, thrombocytopenia (platelet count <150 × 10^9^ /L), or even pancytopenia in severe cases. There is also a well-established predisposition to clonal and malignant myeloid transformation (CMMT), including MDS and AML. A Canadian study indicated that 20% of SDS patients progressed to CMMT by the age of 18 (21). However, patients homozygous for the c.258+2T>C variant appear to have a lower incidence of MDS and AML, potentially due to residual production of functional protein (22, 23). The subjects of this study exhibit a homozygous variant of c.258+2T>C and currently present with neutropenia. Continuous monitoring is essential to evaluate the potential risk of developing MDS and AML. Furthermore, individuals with SDS are at an increased risk of developing various solid tumors, including breast cancer, dermatofibrosarcoma protuberans, esophageal carcinoma, and peritoneal carcinoma (24–26). Pancreatic exocrine insufficiency is another hallmark of SDS, characterized by steatorrhea, malabsorption, and fat-soluble vitamin deficiencies. A fecal elastase level below 100 μg/g is suggestive of exocrine pancreatic insufficiency (5). In this case, the infant presented with constipation shortly after birth, and a fecal elastase level of 70 μg/g indicated pancreatic insufficiency. Although nearly all children with *SBDS* variants show pancreatic insufficiency early in life, approximately 40%–60% may experience improvement in exocrine function with age (27, 28). Elevated aminotransferase levels, the hallmark of liver involvement in SDS, were also observed in this patient at presentation. This manifestation is typically seen in infants and young children, with most achieving normalization of liver enzymes by around 5 years of age (29). Other hepatic abnormalities may include hepatomegaly, fatty infiltration, fibrosis, cholestasis, and cirrhosis (30, 31). To date, no significant abnormalities had been detected on serial liver MRI scans in this child, despite the presence of elevated transaminase levels. The case remains under active surveillance. Skeletal abnormalities in SDS include short stature, skeletal deformities, impaired ossification, and metaphyseal dysplasia (32–34). A study revealed that the 97th percentile of height for SDS patients aged 0–18 years aligns with the 50th percentile of height in the general population (34). However, it had been observed that the child exhibited underdevelopment of the epiphyseal region, with the left femoral head appearing comparatively smaller than the right. The bilateral gluteal folds were symmetric, and the range of motion in both hip joints remained within normal limits. Based on these findings, congenital hip dislocation had been preliminarily ruled out. Common dermatological manifestations of SDS include eczema, ichthyosis, and cutaneous fibrosarcomas (25, 35, 36). Shortly after birth, the child developed eczema-like lesions on both cheeks. Based on the comprehensive clinical presentation and genetic findings, a definitive diagnosis of SDS was established.

It is important to note that the clinical manifestations of SDS overlap with various diseases, which may lead to misdiagnosis. Cystic fibrosis (CF) and SDS are both characterized by upper respiratory tract infections and exocrine pancreatic dysfunction; however, CF is not associated with bone marrow failure (10). Similarly, Pearson syndrome (PS) shares features such as exocrine pancreatic insufficiency and bone marrow dysfunction with SDS, yet PS is seldom inherited and arises primarily from deletions in mitochondrial DNA (37). Given the multisystem complexity of SDS, genetic testing is particularly critical when clinical presentations are ambiguous. Moreover, it provides valuable reference information for the identification of co-occurring rare genetic conditions.

**Figure 2:**
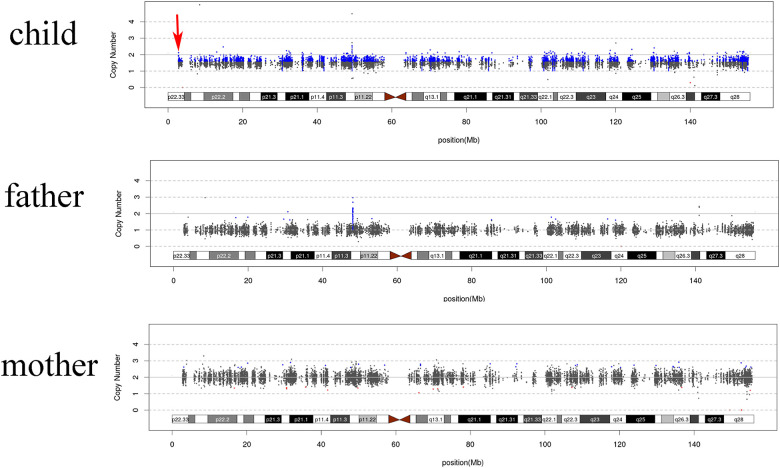
Whole-exome sequencing for copy number variation detection in the proband and their parents.

The genetics of Klinefelter syndrome is defined by the presence of supernumerary X chromosomes, which can result in aberrant expression of X-linked genes or atypical epigenetic modifications, thereby manifesting in the diverse phenotypes associated with different types of Klinefelter syndrome. The supernumerary X chromosomes observed in Klinefelter syndrome may arise from chromosome segregation errors during maternal meiosis I or II, or paternal meiosis I (14). While the majority of human trisomies originate from maternal meiotic nondisjunction, approximately 50% of Klinefelter syndrome cases are attributable to paternal nondisjunction events (38). In this study, the chromosome karyotypes of the child were identified as mosaic 47,XXY/46,XY (Klinefelter syndrome) (Figure 2). This condition may be attributed to either the failure of proper chromosomal segregation during early mitosis in 46,XY fertilized eggs or the loss of an X chromosome during late mitotic stages in 47, XXY (Klinefelter syndrome) fertilized eggs (15).

The clinical manifestations of Klinefelter syndrome are diverse, encompassing aspects of physical growth, language-based learning, executive function, endocrine function, and reproductive health (39). However, the clinical manifestations of patients exhibit significant variation across different age groups. Symptoms and signs associated with chromosomal abnormalities—such as congenital malformations, language-based learning disorders, delayed development of executive functioning, and hypotonia—are predominantly observed prior to puberty(40). In contrast, symptoms and signs related to androgen deficiency, such as sexual dysfunction and oligospermia or azoospermia, are more prevalent during adolescence and adulthood (40). Children presenting with these findings necessitate a differential diagnosis that includes conditions such as azoospermia, hypogonadism, male infertility, and primary testicular failure (41). Patients with the mosaic form of the condition generally exhibit milder clinical manifestations. In this study, the pediatric subject is of the mosaic type. It was observed during infancy that the child had normal testicular size, normal testosterone levels, and no instances of micropenis or congenital anomalies. Nevertheless, as individuals age, certain symptoms may progressively become evident and require vigilant monitoring.

To date, no correlation studies have been conducted between SDS and Klinefelter syndrome. This study represents the first documented case of SDS in conjunction with Klinefelter syndrome in pediatric patients. Both diseases encompass multiple systems and exhibit distinct clinical manifestations. SDS poses a risk of hematological transformation into bone marrow malignancy (5). Moreover, a study indicates that Klinefelter syndrome patients are also at an increased risk of progression to leukemia and lymphoma (42, 43). However, the relationship between the two diseases in terms of certain manifestations, such as those within the hematological system, remains unclear and requires further investigation.

This study presents a systematic analysis of the clinical manifestations and genetic characteristics of a rare case involving SDS co-occurring with Klinefelter syndrome, aiming to enhance pediatricians’ understanding and diagnostic proficiency regarding these two disorders. Allogeneic hematopoietic stem cell transplantation (HSCT) remains the sole curative therapeutic option for SDS patients who have experienced malignant transformation of the bone marrow (44). Moreover, additional symptoms are managed with symptomatic treatment approaches. In this study, the child presenting with granulocytopenia, elevated liver enzymes, and exocrine pancreatic insufficiency were treated with G-CSF to address the granulocytopenia, compound glycyrrhizin for hepatoprotection, and pancreatic enzyme supplements to support pancreatic function. The hallmark clinical feature of Klinefelter syndrome is hypergonadotropic hypogonadism. Consequently, exogenous testosterone supplementation constitutes the cornerstone of therapeutic management (14). In China, it is advisable to initiate exogenous testosterone replacement therapy at the onset of puberty (40). Foreign research has indicated the potential benefits of early hormone therapy (EHT) for infants diagnosed with Klinefelter syndrome (45–47). This therapeutic approach involves administering testosterone during the transient phase known as “mini-puberty,” which occurs in early infancy, with the aim of addressing underlying androgen deficiency (47). This brief period of hormonal development is generally believed to commence approximately two weeks after birth and may persist for at least 24 weeks. EHT is thought to have significant implications for brain development, masculinization processes, and enhancement of language acquisition in affected male infants (45–47). However, recent randomized controlled trials have produced inconclusive results, primarily due to confounding variables. Consequently, there is currently insufficient evidence to definitively ascertain the potential benefits or risks associated with EHT. At present, the testosterone levels of the child under consideration remain within normal reference ranges, with no notable elevations observed in luteinizing hormone or follicle-stimulating hormone. Given concerns regarding possible interactions between EHT and the patient's coexisting genetic conditions, the implementation of EHT is not recommended in this clinical scenario at this time. Ongoing monitoring is essential, and the emergence of clinical indications warrants the prompt initiation of testosterone supplementation.

## Conclusion

SDS and Klinefelter syndrome are both rare disorders that are often misdiagnosed, underdiagnosed, or experience significant diagnostic delays. This paper presents a case study of a pediatric patient diagnosed with two concurrent medical conditions. These findings establish a foundation for the etiological diagnosis, molecular characterization, and genetic counseling of SDS and Klinefelter syndrome. Early identification of these disorders can significantly improve patients’ quality of life and facilitate timely surveillance and management of long-term complications.

## Data Availability

The datasets presented in this article are not readily available because this article is a case report of a single case and involves privacy issues, the data cannot be shared in public database. Requests to access the datasets should be directed to the corresponding author.
